# Case Report: Robotic PeTEP for Totally Extraperitoneal Repair of Incisional Hernia

**DOI:** 10.3389/jaws.2025.14072

**Published:** 2025-02-12

**Authors:** Victor G. Radu

**Affiliations:** Department of General Surgery, Medlife Medical Park Hospital, Bucharest, Romania

**Keywords:** PeTEP, incisional hernia extraperitoneal repair, robotic pre-peritoneal repair, robotic PeTEP, incisional hernia robotic extraperitoneal repair

## Abstract

The development of minimally invasive surgical techniques for ventral hernias has significantly progressed, evolving from IPOM and IPOM + to advancements like eTEP/eTEP-TAR. These newer techniques have demonstrated their effectiveness by delivering excellent postoperative outcomes, despite being introduced less than a decade ago. Recreating traditional procedures, which are considered the “gold standard” in ventral hernia surgery, through minimally invasive methods—such as the Rives-Stoppa repair—could represent the next frontier in abdominal wall surgery. This publication focuses on replicating ventral incisional hernia repair using an extraperitoneal approach, as outlined by Todd Heniford, and taking inspiration from the PeTEP technique for primary ventral hernias, whether associated with diastasis recti or not, as described by Hector Valenzuela.

## Introduction

The minimally invasive surgical treatment of ventral hernias has come a long way, from IPOM by Karl Leblanc [1]and IPOM+ by Jan Kukleta [2], eTEP/eTEP-TAR by Belyansky [3]. The latter techniques, although published less than 10 years ago, have proven their effectiveness through excellent postoperative results [4]. The replication of classical operations, which have become the “gold standard” in ventral hernia surgery, using minimally invasive approaches—such as the Rives-Stoppa [5, 6] procedure—may be the key to advancements in parietal surgery. In this context, replicating the ventral incisional hernia repair using an extraperitoneal approach, as published by Todd Heniford [7]and inspired by the PeTEP approach for primary ventral hernias associated or not with diastasis recti, as described by Valenzuela [8, 9], is the focus of this publication.

## Surgical Technique

In the following, I will present the technique for repairing an M1-M3W2 incisional hernia (considering the EHS classification) [10], using the minimally invasive robotic P-eTEP approach.

**FIGURE 1:**
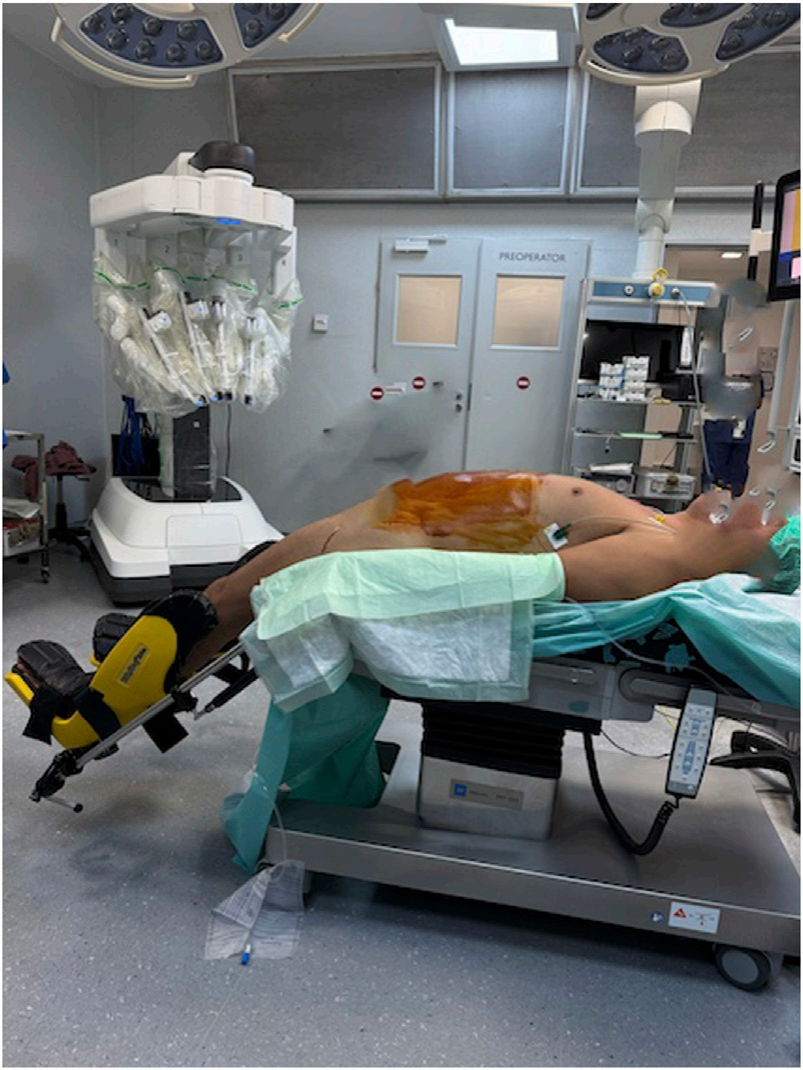
Position of the patient.

Key-stages of the procedure (https://youtu.be/eK1t-ZnADM0):

The positioning of the patient on the operating table is very important. The patient’s trunk should be in hyperextension, with the legs supported on stands, to avoid conflict between the robot arms and the patient’s thighs (Figure 1).

1. Development of the pre-peritoneal space and ports placement.

An optical trocar coupled to an insufflator is used. The insufflation pressure is set to 15 mmHg, and the insufflator is set to maximum flow rate.

Access is achieved by placing the trocar on the midline immediately above the pubic region.

Blunt dissection of the pre-peritoneal space is then performed using the telescope in both the left and right inguinal regions.

**FIGURE 2:**
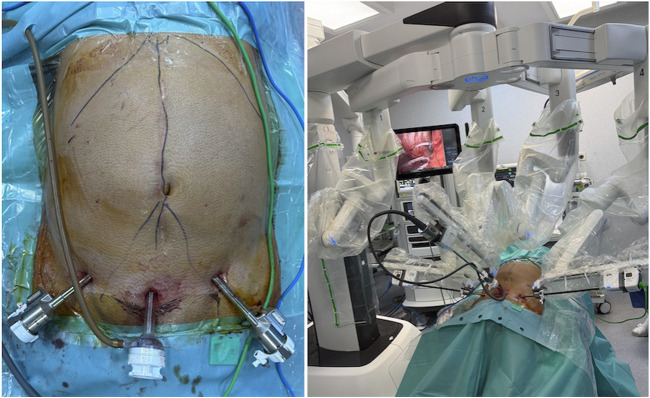
The ports settings.

The working trocars are placed laterally to the inferior epigastric vessels (Figure 2).

2. Dissection of the pre-transversalis/pre-peritoneal space.

**FIGURE 3:**
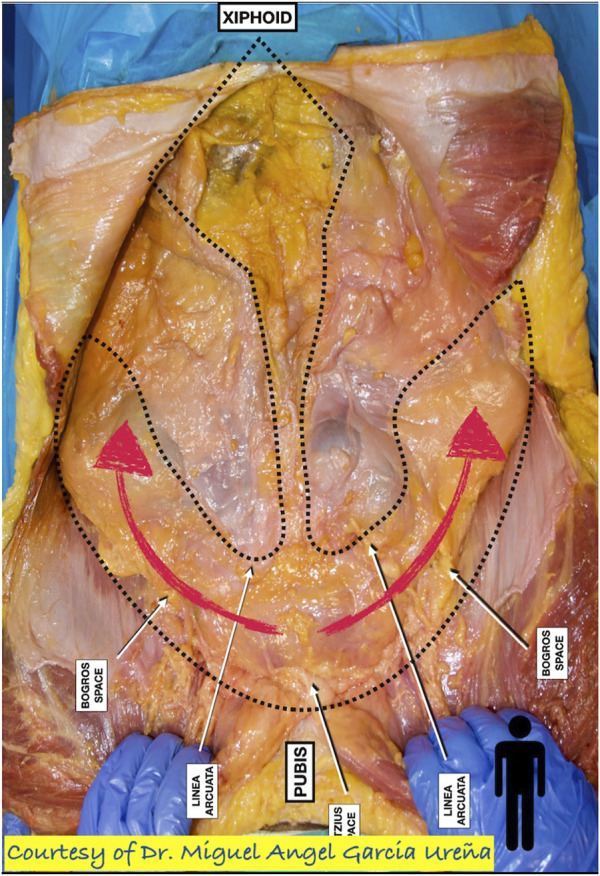
Dissection of the pre-transversalis/pre-peritoneal space (Courtesy of Miguel Angel Ureña).

The dissection begins in the pre-transversalis space, on the flanks, to avoid penetrating the peritoneum. The progression of the initial dissection from the Retzius space in a cranial direction reveals the arcuate line on each side. This approach avoids dissecting the retro-rectus space, as we do in the retro-muscular eTEP approach. The dissection progresses from lateral to medial. Only after achieving the pre-transversalis space on both flanks does the median dissection begin with the reduction of hernia sacs. The goal is to maintain the working space even in the event of peritoneal opening (Figure 3).

Depending on the hernia’s location, the dissection may continue retro-diaphragmatic or retro-xiphoid (for M1 locations) to ensure effective coverage of the defect.

**FIGURE 4:**
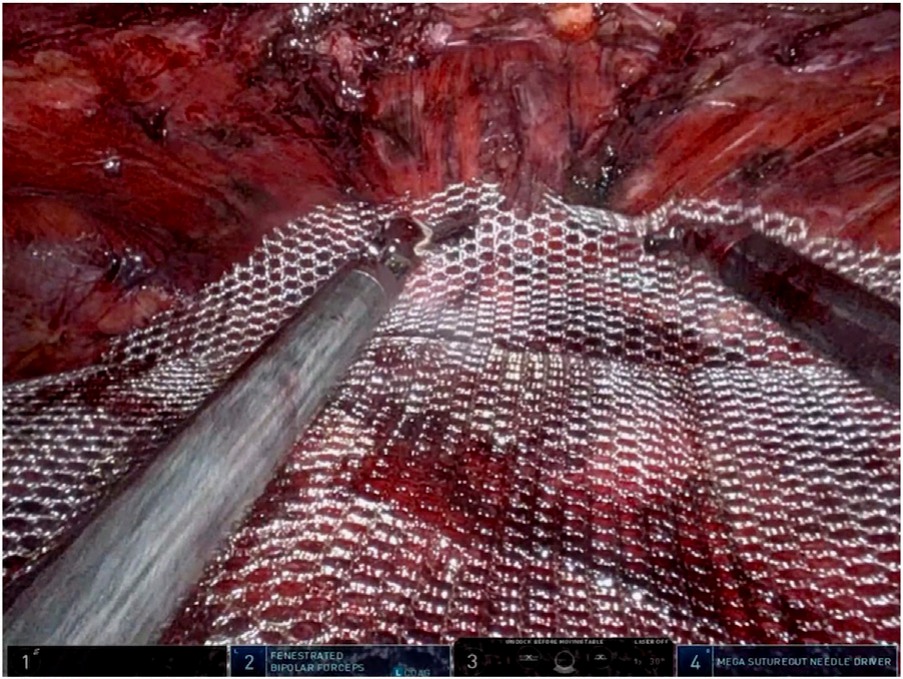
Placement of the mesh.

3. Measurement of the defect and the dissected pre-peritoneal space.
4. Closing the openings in the peritoneum with absorbable 2/0 or 3/0 barbed suture and reconstructing the linea alba.
5. Placement of the mesh/prosthesis. The mesh covers the entire dissected area (Figure 4).

## Results and Discussion

The post-operative course was favorable, with the patient experiencing early active mobilization, reduced pain, and early return of bowel movement. The patient was discharged the day after the surgery, but not more than 20 h postoperatively.

Postoperative follow-up was conducted at 2 weeks and 3 months. The patient did not experience pain or any other issues. Recovery was quick, and they were able to resume physical and sports activities after 1 month without any further problems.

To ensure continued wellbeing and monitor the progress of recovery, it’s advisable for the patient to attend an annual check-up, similar to how all patients operated in our center are monitored.

## Discussion

Repairing ventral incisional hernias using a totally extraperitoneal approach was first published by Todd Heniford. Recently, Hector Valenzuela published a technique for totally extraperitoneal repair of primary ventral hernias, whether associated with diastasis recti or not, using laparoscopic or robotic approaches. The combination of these two principles has led to the possibility of repairing midline incisional hernias using a totally extraperitoneal approach.

The novelty of the procedure lies in the ability to repair ventral hernias without cutting any musculo-fascial structures by dissecting the layers of the abdominal wall—in particular, the pre-peritoneal plane—closing the hernia defect and placing the parietal prosthesis in this space.

An important detail of surgical anatomy that is extremely useful in this procedure is “the fatty trident” — the distribution of the pre-peritoneal fat described by Miguel Angel Ureña [11]. Only by starting the dissection in the pre-transversalis space on the flanks and progressing toward the midline can we maintain the working space even in the situation of opening the peritoneal cavity. The operation continues with the reduction of hernia sacs, closure of any holes in the peritoneum, restoration of the linea alba, and pre-peritoneal parietal prosthesis placement.

Although it is a challenging technique and not easy to reproduce without rigorous training, the preperitoneal approach offers many advantages: the restoration of the abdominal wall architecture without needing to cut any structures (muscles, fascia, etc.). The procedure relies exclusively on the dissection of anatomical planes. This leads to other benefits, such as minimal risk of bleeding or nerve damage. Keeping the peritoneum intact serves as a natural barrier between the mesh and the viscera. As a result, postoperative recovery is quick, with very low levels of pain. The difficulty lies in maintaining the integrity of the peritoneum, which is often very thin and fragile. For this reason, dissection must progress from lateral to medial, as previously described.

The minimally invasive preperitoneal approach to ventral hernias has been recently published. Experience is still limited and cannot yet form the basis of practice guidelines. However, the concept of restoring the abdominal wall architecture as close to normal as possible makes this procedure revolutionary. The limitation is due to the difficulty in dissecting a very thin peritoneum, as well as certain patient-specific particulars, such as previous surgeries in the lower abdominal region, which, through scarring, make extraperitoneal access difficult or impossible.

It should be noted that, in the event of a failure of the preperitoneal dissection, there is a backup plan: the retro-muscular dissection, similar to eTEP. Of course, these aspects will be presented to the patient for informed consent.

## Conclusion

If the minimally invasive replication of the Rives-Stoppa operation and TAR (eTEP/eTEP-TAR) was possible having very good results, an advanced technique, like Todd Heniford’s, which successfully repaired primary and incisional ventral hernias through a pre-peritoneal approach without cutting myofascial structures, has been proven by Hector Valenzuela in primary hernias, laparoscopically and robotically repair, and can be successfully extended to incisional hernias as well.

## Data Availability

The raw data supporting the conclusions of this article will be made available by the authors, without undue reservation.

## Abbreviations

eTEP, Enhanced view of totally extraperitoneal; TAR, transversus abdominis release; PeTEP, pre-peritoneal eTEP; IPOM, intra-peritoneal onlay mesh.
